# Image Segmentation-Guided Visual Tracking on a Bio-Inspired Quadruped Robot

**DOI:** 10.3390/biomimetics11040234

**Published:** 2026-04-02

**Authors:** Hewen Xiao, Guangfu Ma, Weiren Wu

**Affiliations:** 1 School of Aerospace Science, Harbin Institute of Technology, Shenzhen 518055, China; 2 School of Mechanical Engineering and Automation, Harbin Institute of Technology, Shenzhen 518055, China; magf@hit.edu.cn

**Keywords:** visual tracking, quadruped robot, image segmentation, central pattern generator

## Abstract

Bio-inspired quadrupedal robots exhibit superior adaptability and mobility in unstructured environments, making them suitable for complex task scenarios such as navigation, obstacle avoidance, and tracking in a variety of environments. Visual perception plays a critical role in enabling autonomous behavior, offering a cost-effective alternative to multi-sensor systems. This paper proposes an image segmentation-guided visual tracking framework to enhance both perception and motion control in quadruped robots. On the perception side, a cascaded convolutional neural network is introduced, integrating a global information guidance module to fuse low-level textures and high-level semantic features. This architecture effectively addresses limitations in single-scale feature extraction and improves segmentation accuracy under visually degraded conditions. On the control side, segmentation outputs are embedded into a biologically inspired central pattern generator (CPG), enabling coordinated generation of limb and spinal trajectories. This integration facilitates a closed-loop visual-motor system that adapts dynamically to environmental changes. Experimental evaluations on benchmark image segmentation datasets and robotic locomotion tasks demonstrate that the proposed framework achieves enhanced segmentation precision and motion flexibility, outperforming existing methods. The results highlight the effectiveness of vision-guided control strategies and their potential for deployment in real-time robotic navigation.

## 1. Introduction

Quadrupedal robots, owing to their bio-inspired leg structures, exhibit superior locomotion capabilities compared to wheeled robots, making them highly promising for complex task scenarios. Enhancing their ability to autonomously complete tasks necessitates advanced environmental perception. Compared to multi-sensor fusion approaches, such as those relying on LiDAR, visual sensors provide a cost-effective means for robots to perceive their surroundings [1]. Furthermore, the rapid advancement of neural networks has significantly improved computer vision’s ability to extract meaningful information from increasingly complex environments [2]. Among computer vision techniques, image segmentation plays a crucial role as an indispensable auxiliary technology. Its primary function is to decompose visual scenes into meaningful regions, thereby facilitating downstream tasks such as detection, tracking, and recognition. Improving segmentation quality directly enhances the robustness and accuracy of robotic perception systems, with extensive applications in obstacle avoidance [3], navigation [4], and target tracking [5].

Traditional image segmentation methods rely heavily on hand-crafted features or intrinsic priors [6], which often limit their adaptability in complex or cluttered scenes. Recent advances in deep learning, particularly Convolutional Neural Networks (CNNs), have significantly boosted segmentation performance by learning multi-level features from data. However, many CNN-based models still struggle to balance fine-grained detail preservation with global contextual understanding, due to limitations in single-scale feature extraction. Effectively addressing this imbalance requires more sophisticated multi-level and multi-scale representation mechanisms to enhance both spatial resolution and semantic abstraction. To this end, recent Transformer-based approaches employ complex aggregation [7] or dynamic fusion [8] units to provide such global guidance. In contrast, our proposed Global Information Guidance Module (GIGM) achieves similar global-to-local interaction through a more lightweight filter-level sharing strategy, facilitating efficient multi-scale information exchange with significantly reduced structural complexity.

Meanwhile, bio-inspired quadrupedal robots have attracted growing attention for their superior mobility and adaptability in unstructured environments such as rugged terrain or confined spaces. Inspired by animal locomotion, recent work has explored various gait optimization and control strategies. Zhornyak et al. [9] applied genetic algorithms to replicate feline gaits, while Gangapurwala et al. [10] introduced guided constrained policy optimization to generate physically feasible locomotion behaviors. However, these methods often focus on specific motion modes and lack generality when adapting to dynamically changing tasks or environments. As a result, there is a growing need for more flexible and adaptive motion control frameworks that can seamlessly respond to perceptual feedback.

To address challenges in visual perception and motion adaptability, this paper proposes an image segmentation-guided visual tracking framework for bio-inspired quadruped robots.

- On the perception side, we introduce a cascaded neural network equipped with a global information guidance module, which effectively integrates low-level texture details and high-level semantic features across layers, overcoming the limitations of single-scale feature extraction. This design enhances segmentation accuracy, particularly in visually cluttered or blurred environments.
- On the control side, high-level information is incorporated into a biologically inspired central pattern generator (CPG) model to generate coordinated limb and spinal trajectories, enabling comprehensive motion adaptability in dynamically changing conditions. The segmentation results directly inform visual tracking and influence control decisions, creating a closed-loop visual-motor system.
- We conducted extensive evaluations on standard image segmentation datasets and robotic tracking tasks to validate our approach. The results demonstrate that our method outperforms existing approaches in segmentation accuracy and motion flexibility, highlighting its potential for real-time robotic navigation in complex environments.

## 2. Related Work

### 2.1. Image Segmentation

Image segmentation aims to identify regions of greatest interest to people in images. Traditional image segmentation approaches usually predict saliency scores by utilizing hand-crafted cues or intrinsic priors [11,12]. However, they are limited due to their low efficiency and bad generalization ability. With the rise of deep learning, recent methods mostly leverage convolutional neural networks (CNNs) to make a pixel-to-pixel prediction.

Compared with traditional ones, CNN-based methods have shown superior performance on popular image segmentation benchmarks. Among them, early work [13,14,15] mostly adopted an iterative or stage-wise manner to refine the predictions step by step. Some later methods [16,17,18] focus on designing new multi-scale feature-extracting modules and strategies based on the U-shape architecture. Some [19,20,21] introduced various attention mechanisms to enhance the feature representation ability of the network.

In recent years, generative models have rapidly advanced and significantly influenced visual learning tasks, ranging from image synthesis [22] to reinforcement learning [23]. This trend has likewise motivated progress in image segmentation, where researchers have begun to integrate generative paradigms such as VAE-based approaches [24], GAN-driven frameworks [25], and diffusion model-based techniques [26]. These methods leverage generative priors to refine feature representations and promote more stable and coherent segmentation results.

Compared with existing image segmentation methods, we propose a new cascading interaction mode that combines multi-scale information with a global information guidance model to reduce the loss of detailed information and improve accuracy.

### 2.2. Low-Level Gait Controller

Low-level gait control involves managing the robot’s leg movements to maintain balance on various terrains. This includes controlling the swing and support phases of the legs, as well as adjusting the gait cycle and timing. Optimizing low-level gait control is crucial for quadrupedal robots, as it determines their stability, mobility, and efficiency across different environments [27,28]. Controlling the gait of quadruped robots involves multiple parameters and interactions, resulting in a complex parameter space. Efficient computation of gait parameters while maintaining real-time performance with limited computational resources is a challenge.

To tackle this challenge, researchers have proposed various methods in recent years. Some approaches [29,30,31] simplify the gait design of robots using model predictive control. For complex tasks, some methods [32,33] use a hierarchical control structure to match leg movements with torso movements. With the introduction of reinforcement learning, Tsounis et al. [34] use the landing point as a network output, allowing the robot to achieve a more flexible gait. Bellegarda et al. [35] combine CPG with deep reinforcement learning to create adaptable and robust movement patterns.

Unlike the above methods, this paper uses CPGs to parameterize the motion of a crawling quadruped robot, enabling omnidirectional motion across multiple gaits with fewer parameters. At the same time, the method also facilitates the integration with the upper framework.

## 3. Method

**Figure 1:**
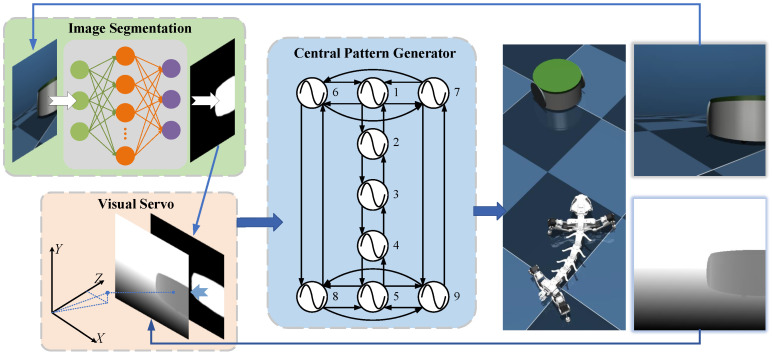
Overview of the proposed visual tracking framework. The on-board camera image is segmented to obtain the target mask; a visual servoing module fuses the mask with depth to estimate target pose and compute a steering command; a CPG-based controller then generates coordinated spine/leg trajectories for tracking. The numbers (1–9) in the Central Pattern Generator (CPG) represent interconnected nodes that facilitate rhythmic signal propagation. The different background colors of the panels (green for Image Segmentation, beige for Visual Servo and blue for CPG) indicate the distinct stages of the processing pipeline.

Figure 1 shows the flowchart of this paper.

The experimental platform used is a crawling quadruped robot with 3 degrees of freedom in its legs and 5 degrees of freedom in its spine, which can only bend and extend in the horizontal plane.

The tracking framework comprises an image segmentation model and a CPG-based low-level controller. The segmentation model takes an RGB image captured by the robot’s camera as input and outputs a binary image that contains only the target. The target position is calculated by combining the distance information obtained from the camera’s internal reference and the depth camera. The desired deflection angle is then calculated based on the target position. The parameters of the CPG are adjusted based on the desired deflection angle to generate mutually coupled rhythmic signals that control the robot’s joint trajectories and foot end, allowing the robot to track the target.

### 3.1. Image Segmentation

#### 3.1.1. Cascaded Information Interaction Network

To precisely segment the target and facilitate the visual servoing module in calculating its position, we use the Swin transformer [36] as an encoder because of its unique advantages: the Swin transformer incorporates a local attention mechanism, inherits the advantages of CNNs in processing large images, and uses a window-based approach to exploit the transformer’s capabilities in long-range dependency modeling. To extract scale-specific features based on different backbone networks, we introduce an additional convolutional layer with a kernel size of 1 to standardize the channel dimensions. Consequently, the resulting unified channel features can be denoted as $E=\{E_{i},1\leq i\leq I\}$, where *I* is typically set to 5.

**Figure 2:**
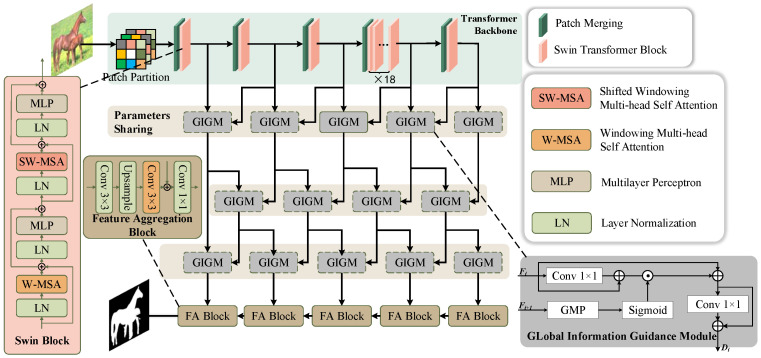
Overview of the proposed segmentation network. A Swin-Transformer encoder extracts multi-scale features, which are progressively fused via cascaded cross-resolution interactions. GIGM uses higher-level context to calibrate lower-level features, and FA blocks refine the representations to produce the final saliency map.

As shown in Figure 2, after applying convolutional pooling for down-sampling and subsequent up-sampling to restore the original resolution, images often suffer from blurring and loss of fine details. The conventional approach involves cascading feature maps at the same resolution along both the bottom-up and top-down paths, which mitigates the loss of local features to some extent. However, a direct feature extraction approach may limit multi-scale information fusion, as hierarchical feature interactions are often underutilized. To overcome this constraint, we propose a Cascaded Information Interaction Network, which enables multi-scale information exchange at the filter level. This technique establishes a structured mechanism for progressive feature refinement, ensuring effective communication across different resolution layers. Additionally, we recognize that deep architectures typically yield enhanced performance due to their ability to model complex patterns. Building on this idea, we expand the interaction layers in our model to strengthen hierarchical feature representation. Given the channel unified feature maps from the encoder E, the features delivered to the decoder $D=\{D_{i},1\leq j\leq J\}$ could be obtained by cascaded interactors as

(1) $$D_{j}=F^{q}\left(E_{k},\ldots ,E_{m}\right),\;1\leq j\leq 5,\;1\leq k\leq m\leq 5$$

where F denotes the feature fusion in each interaction level, *q* indicates the number of function actions, which means the number of cascading levels.

#### 3.1.2. Global Information Guidance Module

In segmentation tasks, an efficient multiscale module significantly enhances module performance. Higher-level information can serve to guide and enhance the interaction of lower-level information across different scales. To maintain the compression of both local and relative global information, we introduce a global information guidance module (GIGM). The higher-level information can serve to guide the lower-level information, thereby enhancing the interaction between different scales of information. The module input contains the lower-level information $F_{i}$, which has been processed by a 1×1 convolutional layer. In addition, the higher-level information, $F_{i+1}$, has been subjected to Global Maximum Pooling (GMP) and sigmoid function, as shown by the gray box in Figure 2. The higher-level information is compressed to calibrate the lower-level information, thereby preserving local features. Finally, the output $D_{i}$ is obtained after a 1×1 convolutional layer. $D_{i}$ serves as an information guide from the relatively higher level pathway to the lower level pathway. The module is expressed as follows:

(2) $$G_{i+1}=Sigmoid\left(GMP\left(F_{i+1}\right)\right),1\leq i\leq M-1$$

(3) $$D_{i}=\left(Conv^{1}+1\right)\left(G_{i+1}⊙\left(Conv^{1}+1\right)\left(F_{i}\right)+F_{i}\right),\;1\leq i\leq M.$$

### 3.2. Visual Servo Controller

To transform the target binary map generated by the segmentation model into a tracking instruction for the robot, we combine depth measurements to obtain distance information and calculate the desired deflection angle.

The zero-order moments ($M_{00}$) and first-order moments ($M_{10},\;M_{01}$) are solved for the binary map to obtain the position of the center-of-mass pixel point of the target object, respectively.

(4) $$M_{00}=\sum_{W}\sum_{H}P\left(w,h\right),\;M_{10}=\sum_{W}\sum_{H}w\cdot P\left(w,h\right),\;M_{01}=\sum_{W}\sum_{H}h\cdot P\left(w,h\right)$$

P(w,h) has only two values, 0 (black) or 1 (white), and therefore $M_{00}$ represents the sum of the target regions in the map. $M_{10}$ and $M_{01}$ represent the accumulation of w and h coordinate values of the target area, respectively. Therefore, the center of mass position of the target in the figure is $w_{c}=\frac{M_{10}}{M_{00}},\;h_{c}=\frac{M_{01}}{M_{00}}$.

**Figure 3:**
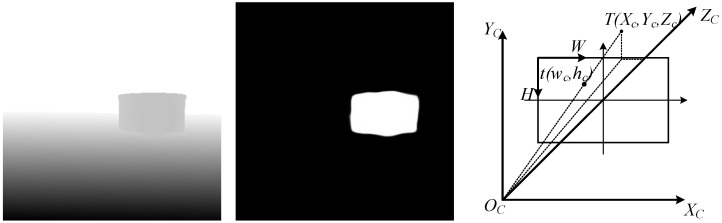
Desired deflection angle calculation.

The depth map is used to obtain distance information corresponding to the pixel point of the center of mass. This is combined with the imaging principle of the camera to solve the position of the target center of mass under the camera coordinate system. The image coordinate system (W, H) is defined in Figure 3 with the upper left corner of the image as the origin. The coordinate origin is the optical center position of the camera. The X-axis and Y-axis are parallel to the W-axis and H-axis of the image coordinate system, and the Z-axis is the optical axis of the camera’s camera coordinate system. The position of the target center of mass in the image under the camera system can be obtained from the imaging principle of the camera by the following equation:

(5) $$\left\{\begin{array}{c} X_{c}=\frac{\left(i-w_{0}\right)}{f_{x}}*D\left(i,j\right)\\ Y_{c}=\frac{\left(j-h_{0}\right)}{f_{y}}*D\left(i,j\right)\\ Z_{c}=D\left(i,j\right) \end{array}\right.$$

The depth value of pixel point (i, j), designated as D(i, j). (fx, fy, w0, h0) represent internal camera parameters. The desired angle of turn in the horizontal direction of the quadrupedal robot is $\phi =\arctan(\frac{\mathrm{Xc}}{\mathrm{Zc}})$.

### 3.3. CPG-Based Low-Level Gait Control

The coupling of leg and spinal motion is controlled using CPG. It is designed to mimic the central nervous system of an organism, enabling quadrupedal robots to autonomously generate gaits and adapt to different terrains and environments.

The connection scheme and functional allocation of the oscillators of the CPG are illustrated in Figure 1. The phase expression for the cpg oscillator is given by (6).

(6) $$\begin{array}{cc} \; & \dot{\theta_{i}}=2\pi *f+\sum_{j}\left(\omega_{ij}\sin\left(\theta_{j}-\theta_{i}-\vartheta_{ij}\right)\right) \end{array}$$

where $\theta_{i}$ is the phase of the oscillator and *f* is the robot walking step frequency. The coupling between the oscillators is realized by the weights $\omega_{ij}$ and the phase difference $\vartheta_{ij}$. The value of the phase difference is determined by the gait of the robot.

The robot’s spine is controlled by the oscillators 1–5, and the actual angle sent to the spinal joints is obtained by combining the amplitude and bias based on the phase calculated above, which is calculated as (7)

(7) $$\begin{array}{cc} \; & {\dot{r}}_{i}^{sp}=a_{r}\left(R_{i}^{sp}-r_{i}^{sp}\right)\\ \; & \dot{x_{i}}=a_{x}\left(X_{i}-x_{i}\right)\\ \; & \phi_{i}=x_{i}+r^{sp}\cos\left(\theta_{i}\right) \end{array}$$

where $r_{i}^{sp}$ and $x_{i}$ are state variables denoting the amplitude and bias of the oscillator, respectively, and $R_{i}^{sp}$ and $X_{i}$ are the desired amplitude and desired bias of the oscillator, and the rate of convergence of the oscillator’s amplitude and bias is expressed in terms of positive gains $a_{r}$ and $a_{x}$. When the robot steers, the spine is driven to bend in the desired direction by the bias term $X_{i}$. The bias $X_{i}$ is calculated from the visual servo controller output $\psi_{i}$. We take 4 maps at equal intervals during a gait cycle and find the bias by counting the distribution of pixel points.

The CPG parameters (e.g., the gains $a_{r}$ and $a_{x}$) were selected via manual tuning. We started from stable baseline values and then iteratively adjusted the parameters in simulation to (i) maintain stable oscillations, (ii) ensure smooth convergence of amplitude/bias without overshoot, and (iii) achieve low tracking error while avoiding foot slippage and excessive joint excursions. The final parameter set was chosen based on the best overall trade-off across these criteria.

**Figure 4:**
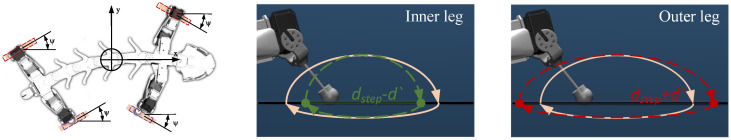
Robot turning motion.

In this paper, we utilize 6th to 9th oscillators to facilitate the generation of foot trajectories. Subsequently, the joint commands are derived through inverse kinematics. To align the oscillator state with the selected gait, we transform the oscillator phase by the ratio of the gait support phase to the swing phase to obtain $\theta^{\prime }$. Figure 4 illustrates the spine posture and foot trajectory of the robot during a turn. The coordinates of the foot end position are as follows:

(8) $$\begin{array}{cc} {\bar{p}}_{i,x} & =-\left(d_{\mathrm{step}\;}\pm d\right)\cos\left(\theta_{i}^{\prime }\right)\cos\left(\psi_{i}\right)\\ {\bar{p}}_{i,y} & =-\left(d_{\mathrm{step}\;}\pm d\right)\cos\left(\theta_{i}^{\prime }\right)\sin\left(\psi_{i}\right) \end{array}$$

(9) $${\bar{p}}_{i,z}=\left\{\begin{array}{cc} -h+g_{c}\sin\left(\theta_{i}^{\prime }\right) & \;\mathrm{if}\;\alpha_{i}\in \left[0,\frac{\pi }{2}\right)\\ -h+g_{p}\sin\left(\theta_{i}^{\prime }\right) & \;\mathrm{otherwise}\; \end{array}\right.$$

where $g_{c}$ is the maximum ground clearance achieved during the swing phase, while $g_{p}$ denotes the maximum ground penetration attained during the stance phase. The step length and robot height are denoted as $d_{step}$ and *h*, respectively. The compensation amount *d* is related to the steering angle. The plus and minus signs, respectively, indicate the outer foot trajectory and the inner foot trajectory.

## 4. Experimental Results

### 4.1. Image Segmentation

#### 4.1.1. Experimental Setup

The evaluation datasets utilized in our study include five well-established datasets: ECSSD [37], PASCAL-S [38], DUT-OMRON [39], HKU-IS [40], and DUTS-TE [41]. For model training, we consistently employ the DUTS-TR dataset [41] across all experiments, following established practices in image segmentation research.

Our model was trained for 60 rounds in batches of 30, and we selected the optimizer with a learning rate of 0.005, momentum of 0.9, and weight decay of $5\times 10^{-5}$. The image input size was resized to 384 × 384 for both training and testing. The detailed hyperparameters and preprocessing steps are summarized in Table 1.

To assess the effectiveness of various methods, we utilize three commonly used metrics: the F-measure score ($F_{\beta }$), the mean absolute error (MAE), and the S-measure score ($S_{\alpha }$). ($F_{\beta }$) is calculated as follows:

(10) $$F_{\beta }=\frac{(1+\beta^{2})\times \mathrm{Precision}\times \mathrm{Recall}}{\beta^{2}\times \mathrm{Precision}+\mathrm{Recall}}.$$

To impose a higher weight for accuracy, we set $\beta^{2}$ to 0.3. At the pixel level, MAE evaluates the average absolute difference between the predicted image *P* and the labeled image *L*.

(11) $$\mathrm{MAE}=\frac{1}{W\times H}\sum_{x=1}^{W}\sum_{y=1}^{H}\left|P\left(x,y\right)-L\left(x,y\right)\right|,$$

where the width and height of the image are denoted by *W* and *H*, respectively. The S-measure ($S_{\alpha }$) integrates both object-aware ($S_{o}$) and region-aware ($S_{r}$) structural similarity components, and is calculated as follows:

(12) $$S_{\alpha }=\gamma S_{o}+\left(1-\gamma \right)S_{r},$$

where γ is 0.5 as is commonly done.

The loss function utilized in this paper combines an intersection-over-union (IoU) loss with a binary cross-entropy loss (BCE): $l=l_{iou}+l_{bce}$. Because of its excellent robustness, the binary cross-entropy (BCE) loss function is widely used in binary classification and is obtained by calculating the pixel-by-pixel loss of the image:

(13) $$l_{bce}\left(p,l\right)=-\frac{1}{n}\sum_{k=1}^{n}\left[l_{k}\log\left(p_{k}\right)+\left(1-l_{k}\right)\log\left(1-p_{k}\right)\right]$$

*p* and *l* stand for the predicted image and label, respectively. *k* is the index of the pixel and *n* is the number of pixels in *x*. In contrast to the BCE loss function, which emphasizes differences at the pixel level, the IoU loss considers the overall graph similarity, and its definition is as follows:

(14) $$l_{\mathrm{iou}\;}\left(p,l\right)=1-\frac{\sum_{k=1}^{n}\left(l_{k}*p_{k}\right)}{\sum_{k=1}^{n}\left(l_{k}+p_{k}-l_{k}*p_{k}\right)}.$$

#### 4.1.2. Comparisons to the State of the Art

We compared the proposed image segmentation method with 22 state-of-the-art approaches, including PAGR [20], DGRL [13], PiCANet [19], MLMS [42], PAGE [21], ICTB [14], CPD [15], BASNet [43], PoolNet [16], CSNet [44], GateNet [17], MINet [45], ITSD [46], VST [47], MSFNet [48], CII [49], PoolNet+ [50], DCN [51], DNA [52], RCSB [53], PriorNet [7], and NASAL [54]. To ensure a fair comparison, we either utilize saliency maps shared by the authors or compute their released models. We then quantitatively compare the obtained results by calculating the F-measure score $F_{\beta }$, the S-measure score $S_{\alpha }$ and the mean absolute error (MAE) of our method alongside the other methods. Table 2 presents the results of the other advanced measurement methods mentioned. On the ECSSD dataset, our method achieves the highest $F_{\beta }$ (0.952) and the lowest MAE (0.028), while maintaining a high $S_{\alpha }$ value of 0.933. These results suggest enhanced capacity for capturing fine details and complex object structures, particularly in cluttered scenes. Similarly, on PASCAL-S, our model maintains leading performance, with minimized MAE and competitive $F_{\beta }$ and $S_{\alpha }$ values, indicating improved robustness in handling occlusion and challenging backgrounds. Performance on HKU-IS further highlights the model’s generalization capabilities, recording an $F_{\beta }$ of 0.898, MAE of 0.031, and $S_{\alpha }$ of 0.929, surpassing comparative methods across all metrics. On more challenging datasets such as DUT-OMRON and DUTS-TE, our method maintains its advantages. It shows significant improvement of 1.1% and 0.8% compared with the famous PoolNet+ model [50], which confirms its effectiveness in delineating object boundaries under complex scenes. Our model achieves leading performance in salient object detection, owing to its unique architecture that combines multi-scale feature interaction with global information guidance. This design enhances detail preservation while maintaining accurate global context.

**Figure 5:**
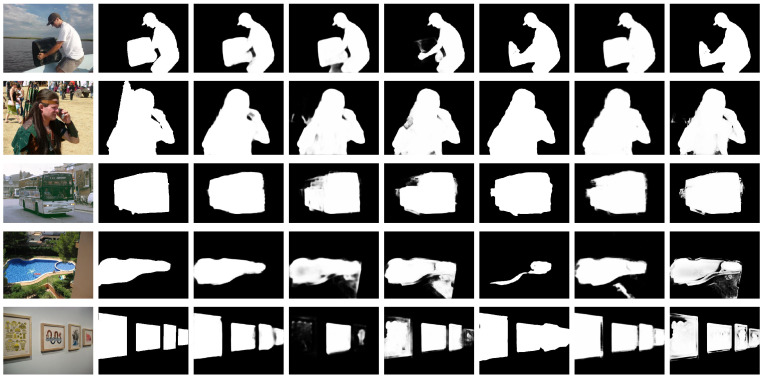
Visual comparison of saliency maps with state-of-the-art methods. From left to right: Input image, Ground truth, Ours, DNA, CII, MSFNet, VST and ITSD. Our approach consistently produces the best results.

In Figure 5, we present example saliency maps generated by our method. The input images used for the visual comparison are taken from standard datasets (not from the simulation camera). These maps demonstrate our method’s ability to produce accurate results with clear boundaries and uniform highlights.

#### 4.1.3. Speed Analysis

In this speed analysis experiment, we evaluate inference latency when processing a 384×384 input image on two hardware platforms (one station with a single NVIDIA RTX-4090 GPU and one laptop with a single NVIDIA RTX-2060 GPU). Our method is built by adding lightweight modules on top of the FPN baseline, and we use this FPN implementation as the direct baseline for comparison. Although the proposed method achieves substantially better segmentation and tracking performance in the previous subsection, it introduces only a marginal inference overhead over FPN (about 1.74 ms on the station and 1.11 ms on the laptop, as shown in Table 3). This result indicates that the performance gains are obtained with minimal additional computational cost on both devices. Taken together, the strong perception accuracy and low computational overhead suggest that the proposed model is well suited for practical deployment in real-world robotic navigation tasks.

### 4.2. Low-Level Controller

In this paper, we use the Mujoco as the simulator. The robot physical parameters (including the 3-DOF legs and the 5-DOF spine) and the MuJoCo simulation configuration follow the setup reported by Horvat et al. [57]. To assess the effectiveness of this method in improving robot locomotion, we compared it with the spineless control and open-loop methods of tracking the straight line and the sine line.

**Figure 6:**
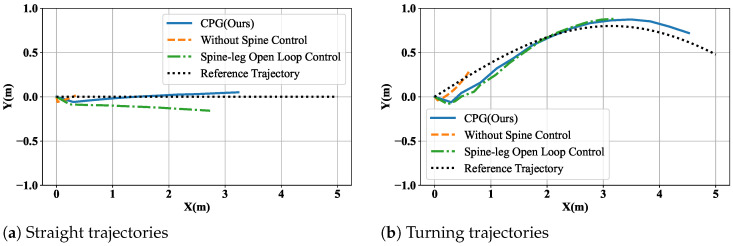
Comparison of motion performance.

Here, the open-loop control method applies trigonometric signals to the spine joints; the signals are the same as the step frequency. We evaluated the robot’s agility by letting it walk the same number of steps under different methods. Figure 6a shows the experimental results. During the experiment, the robot walked for 10 gait cycles while tracking a straight-line trajectory. The results indicate that the robot’s motion performance is poor without spine coordination (orange line). However, open-loop spine control (green line) greatly improves the motion performance, resulting in a travel distance of more than 2.5m. The method proposed in this paper (blue line) further increases the travel distance and significantly reduces the tracking error compared with open-loop control.

To compare turning ability across methods, the robot tracked a segment of a sinusoidal trajectory while taking the same number of steps. The experimental results in Figure 6b indicate that the spineless control method (orange line) performs poorly in coordination and in tracking the target trajectory. Similarly, the open-loop method (green line) does not capture the interaction between the spine and the legs, resulting in larger errors at large steering angles. Our method (blue line) exhibits superior motion performance, advancing farther while taking the same number of steps. Furthermore, the tracking error is smaller, particularly during the (0,2m) convergence phase towards the target trajectory. This demonstrates the effectiveness of our method in improving the locomotion ability of the robot.

### 4.3. Visual Tracking

To validate the performance of the visual tracking framework presented in this paper, we utilize a moving Epuck robot as the tracking target.

**Figure 7:**
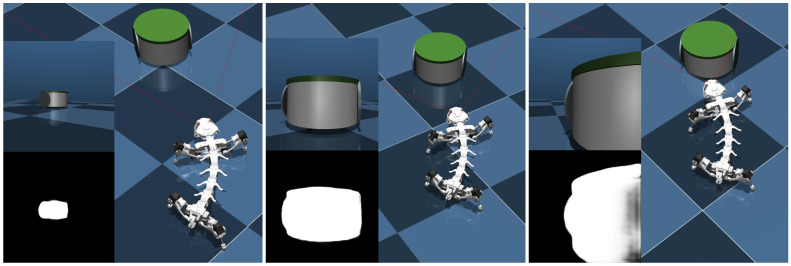
Simulation scenario of quadrupedal robot tracking task.

In the simulation, the Epuck robot moves at a speed of 0.5 m/s, while the quadrupedal robot continuously captures and tracks it using visual input. The simulation scenario for the tracking experiment is illustrated in Figure 7. The quadrupedal robot gradually reduces the distance to the target robot, eventually catching up and maintaining the track.

**Figure 8:**
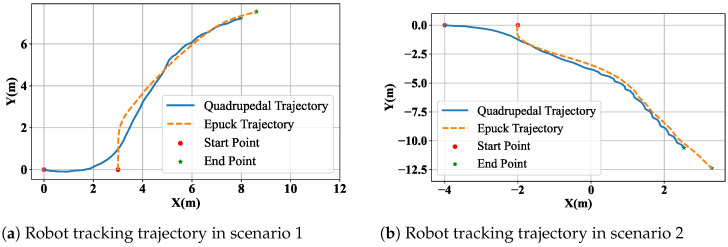
Robot tracking performance.

As illustrated in Figure 8a, the quadrupedal robot is positioned at the origin to track the Epuck robot at (2.5, 0) and approaches the Epuck robot to within 0.5 m. At the initial stage, the Epuck robot moves in the positive direction of the *Y*-axis, while the quadrupedal robot moves in the positive direction of the *X*-axis.

There is a significant difference in the direction of motion between the two. According to the instructions of the vision-tracking framework, the quadrupedal robot narrows the deviation and successfully tracks the Epuck robot’s trajectory. The quadrupedal robot was again tasked with continuously tracking the target, as illustrated in Figure 8b. The tracking framework successfully enabled the quadrupedal robot to capture the target and track its trajectory. The target also altered its direction of motion along the way, and the tracking framework provided the correct instructions for the quadrupedal robot to make timely adjustments.

Throughout the experiment, the target robot altered its direction of motion multiple times. The tracking framework provided the quadrupedal robot with the necessary instructions to make timely adjustments, ensuring continuous and accurate tracking of the Epuck robot’s trajectory. This demonstrated the robustness and effectiveness of the visual tracking framework in dynamic and changing environments.

## 5. Discussion

One critical consideration for the proposed framework is its applicability to real-world environments, given that the current validation is conducted within the MuJoCo simulation. However, it is important to note that the visual perception module (the cascaded transformer-based network) is trained on large-scale, standard real-world datasets. These datasets encompass a wide spectrum of environmental challenges, such as drastic lighting variations, complex shadows, and diverse surface textures, which are common in unconstrained real-world scenarios. By learning from these diverse real-world samples, the model develops high-level semantic understanding and boundary-awareness that are inherently robust to the environmental factors mentioned. While the MuJoCo simulator provides a controlled environment for testing the closed-loop gait adaptation logic, the perception-action backbone is built upon real-world visual features, significantly bridging the gap between simulation and reality. Future work will further explore the Sim-to-Real transition by deploying the system on physical quadrupedal platforms to account for complex hardware-level dynamics and sensor noise.

## 6. Conclusions

In this work, we present a visual tracking control framework for quadrupedal robots. A cascade interaction network is introduced to enhance the information interaction ability, which improves the accuracy and efficiency of the image segmentation model. Furthermore, we convert the segmented target image into the desired deflection angle of the quadruped robot. For low-level gait control, this paper introduces a central pattern generator to parameterize quadruped motion and upper-level commands, enabling flexible switching between different motion modes. The effectiveness of the image segmentation proposed in this paper is verified through experiments and comparisons on standard datasets. And we verify the value of the framework in quadrupedal robot tasks.

## Figures and Tables

**Table 1:** Training hyperparameters and data preprocessing.

Item	Setting
Training epochs (rounds)	60
Batch size	30
Optimizer	SGD
Initial learning rate	0.005
Learning rate schedule	Fixed (no decay)
Momentum	0.9
Weight decay	$5\times 10^{-5}$
Input resize	384 × 384 (train/test)
Normalization	Standard dataset normalization
Data augmentation	None

**Table 2:** Comparisons of our method with other state-of-the-art methods on five popular SOD benchmarks.

Method	ECSSD			PASCAL-S			DUT-OMRON			HKU-IS			DUTS-TE		
	$F_{\beta }$ ↑	**MAE**↓	$S_{\alpha }$↑	$F_{\beta }$ ↑	**MAE**↓	$S_{\alpha }$↑	$F_{\beta }$ ↑	**MAE**↓	$S_{\alpha }$↑	$F_{\beta }$ ↑	**MAE**↓	$S_{\alpha }$↑	$F_{\beta }$ ↑	**MAE**↓	$S_{\alpha }$↑
PAGR [20]	0.927	0.061	0.889	0.847	0.089	0.822	0.771	0.071	0.775	0.919	0.047	0.889	0.854	0.055	0.839
DGRL [13]	0.922	0.041	0.903	0.844	0.072	0.836	0.774	0.062	0.806	0.910	0.036	0.895	0.828	0.049	0.842
PiCANet [19]	0.935	0.047	0.917	0.864	0.075	0.854	0.820	0.064	0.830	0.920	0.044	0.904	0.863	0.050	0.868
MLMS [42]	0.930	0.045	0.911	0.853	0.074	0.844	0.793	0.063	0.809	0.922	0.039	0.907	0.854	0.048	0.862
PAGE [21]	0.931	0.042	0.912	0.848	0.076	0.842	0.791	0.062	0.825	0.920	0.036	0.904	0.838	0.051	0.855
ICTB [14]	0.938	0.041	0.918	0.855	0.071	0.850	0.811	0.060	0.837	0.925	0.037	0.909	0.855	0.043	0.865
CPD [15]	0.939	0.037	0.918	0.859	0.071	0.848	0.796	0.056	0.825	0.925	0.034	0.907	0.865	0.043	0.869
BASNet [43]	0.942	0.037	0.916	0.857	0.076	0.838	0.811	0.057	0.836	0.930	0.033	0.908	0.860	0.047	0.866
PoolNet [16]	0.944	0.039	0.921	0.865	0.075	0.850	0.830	0.055	0.836	0.934	0.032	0.917	0.886	0.040	0.883
CSNet [44]	0.944	0.038	0.921	0.866	0.073	0.851	0.821	0.055	0.831	0.930	0.033	0.911	0.881	0.040	0.879
GateNet [17]	0.946	0.040	0.920	0.877	0.068	0.858	0.831	0.055	0.838	0.935	0.033	0.915	0.889	0.040	0.885
MINet [45]	0.947	0.034	0.925	0.874	0.064	0.856	0.826	0.056	0.833	0.936	0.028	0.920	0.888	0.037	0.884
ITSD [46]	0.947	0.035	0.925	0.871	0.066	0.859	0.823	0.061	0.840	0.933	0.031	0.916	0.883	0.041	0.885
VST [47]	0.951	0.034	0.932	0.875	0.062	0.872	0.829	0.058	0.850	0.942	0.030	**0.929**	0.891	0.037	0.896
MSFNet [48]	0.943	0.033	0.915	0.865	0.061	0.852	0.824	0.050	0.832	0.930	0.027	0.909	0.881	0.034	0.877
CII [49]	0.950	0.034	0.926	0.882	0.062	0.865	0.831	0.054	0.839	0.939	0.029	0.920	0.890	0.036	0.888
PoolNet+ [50]	0.949	0.040	0.925	0.879	0.068	0.864	0.831	0.056	0.842	0.941	0.034	0.921	0.894	0.039	0.890
DCN [51]	0.952	0.031	0.928	0.872	0.062	0.861	0.823	0.051	0.845	0.940	0.027	0.922	0.894	0.035	0.891
DNA [55]	0.940	0.043	0.915	0.855	0.079	0.837	0.803	0.063	0.818	0.927	0.036	0.905	0.873	0.046	0.860
RCSB [53]	0.945	0.033	0.922	0.879	0.059	0.860	**0.849**	**0.049**	0.835	0.939	0.027	0.918	0.897	0.035	0.881
PriorNet [7]	**0.953**	0.031	0.931	0.881	0.059	0.869	0.839	0.051	0.849	0.940	0.029	0.920	**0.901**	0.033	0.897
NASAL [54]	0.925	0.052	0.904	0.836	0.092	0.825	0.800	0.069	0.818	0.913	0.044	0.898	0.833	0.060	0.841
Ours	**0.952**	**0.028**	**0.933**	**0.888**	**0.054**	**0.879**	**0.842**	**0.049**	**0.858**	**0.943**	**0.025**	**0.929**	**0.898**	**0.031**	**0.900**

Note: The “Ours” row is highlighted in bold to emphasize the performance of the proposed method in this study. The arrows (↑) next to the relevant metrics indicate that higher values are better, whereas the arrows (↓) indicate that lower values are better.

**Table 3:** Comparison of inference latency between the proposed network and FPN on different devices.

Method	Station	Laptop
FPN [56]	13.48 ms	44.15 ms
**Proposed**	15.22 ms	45.26 ms

## Data Availability

The data presented in this study are available on request from the corresponding author.
